# Unveiling *Ralstonia* spp. in the Neonatal Intensive Care Unit: Clinical Impacts and Antibiotic Resistance

**DOI:** 10.3390/antibiotics14030259

**Published:** 2025-03-03

**Authors:** Julia Burzyńska, Aleksandra Tukendorf, Marta Fangrat, Katarzyna Dzierżanowska-Fangrat

**Affiliations:** 1 Department of Clinical Microbiology and Immunology, The Children’s Memorial Health Institute, Aleja Dzieci Polskich 20, 04-730 Warsaw, Poland; j.burzynska@ipczd.pl (J.B.); a.tukendorf@ipczd.pl (A.T.); 2 Maria Sklodowska-Curie Medical Academy, Aleja Solidarności 12, 03-411 Warsaw, Poland; marta.fangrat@uczelniamedyczna.com

**Keywords:** *Ralstonia mannitolilytica*, outbreak, resistance

## Abstract

**Background/Objectives:***Ralstonia* spp., opportunistic Gram-negative bacilli, pose increasing risks for nosocomial infections, particularly in neonatal intensive care units (NICUs). This study investigates an outbreak caused by *Ralstonia mannitolilytica* in an NICU during the COVID-19 pandemic, examining colonization and infection risk factors, clinical outcomes, and antibiotic resistance. **Methods**: A retrospective analysis was conducted on neonates hospitalized in a tertiary NICU from 2020 to 2021. Colonization and infection were identified via microbiological testing of clinical samples. Risk factors, such as mechanical ventilation, vascular access, mode of feeding, and antibiotic use, were recorded. Environmental sampling identified potential contamination sources. Antibiotic susceptibility was determined using EUCAST PK/PD breakpoints. **Results**: Among 36 neonates affected, 31 were colonized, and 5 developed infections, including bloodstream infection, pneumonia, surgical site infection, and urinary tract infection. Environmental investigations revealed contaminated water heaters as the primary sources. All isolates showed resistance to carbapenems and aminoglycosides but retained susceptibility to trimethoprim-sulfamethoxazole. The vast majority were susceptible to fluoroquinolones. **Conclusions**: This outbreak underscores the role of environmental water reservoirs, invasive procedures, and broad-spectrum antibiotics in *R. mannitolilytica* colonization and infection. Biofilm formation and antibiotic resistance complicate eradication and treatment. Heightened surveillance, rigorous infection control, and antimicrobial stewardship are crucial for mitigating risks in NICU settings.

## 1. Introduction

Neonatal sepsis, defined as a life-threatening and dysregulated inflammatory response to bloodstream infection in infants of less than 28 days, remains a leading cause of morbidity and mortality in neonatal intensive care units (NICUs), particularly among preterm and extremely low birth weight (ELBW) infants [1]. These neonates are uniquely susceptible to infections due to their underdeveloped immune systems, reliance on invasive medical devices, and extended hospital stays. While common pathogens such as *Escherichia coli*, *Klebsiella pneumoniae*, Group B *Streptococcus*, and staphylococci dominate the clinical landscape of neonatal sepsis—especially early-onset sepsis occurring within the first 72 h of life—the emergence of rare and opportunistic pathogens poses new challenges for clinicians [1,2]. These less common pathogens are more frequently associated with late-onset sepsis, which develops after the initial 72-h period [1]. Among them, *Ralstonia* species have recently drawn increasing attention as significant nosocomial threats [3,4].

*Ralstonia* spp. are aerobic, Gram-negative, non-fermenting bacilli that thrive in water and moist environments [5]. Traditionally regarded as low-virulence organisms, they have demonstrated a remarkable ability to persist in healthcare settings, particularly in medical equipment and solutions [6]. Two species, *Ralstonia mannitolilytica* and *Ralstonia pickettii*, have been implicated in a range of healthcare-associated infections, including bacteremia and sepsis [5,6]. The clinical importance of these pathogens is magnified by their resistance to multiple antibiotics and their association with outbreaks linked to contaminated medical devices and pharmaceutical products.

A few outbreaks of *Ralstonia* infections have been reported in pediatric and neonatal populations. In 2001, an outbreak of *R. pickettii* bacteremia in a California NICU was traced to contaminated heparin flushes prepared in the hospital pharmacy [7]. This outbreak, which involved 18 neonates, highlighted the vulnerability of NICU patients to pathogens introduced via routine medical interventions. Similarly, *R. pickettii* bacteremia was observed in pediatric patients receiving extracorporeal membrane oxygenation (ECMO) therapy in a Canadian hospital, where temperature-control units of ECMO systems were identified as the environmental reservoir [8]. A particularly concerning outbreak of *R. mannitolilytica* occurred in 2005, when 38 pediatric patients in 12 U.S. states were infected via contaminated oxygen delivery devices [9]. Investigation revealed intrinsic contamination of these devices, with inadequate disinfection protocols failing to eliminate the pathogen.

Here, we describe an outbreak caused by *R. mannitolilytica* in the neonatal intensive care unit (NICU) in a tertiary pediatric hospital during the COVID-19 pandemic (2020–2021). Risk factors for *R. mannitolilytica* colonization/infection, environmental reservoirs of this microorganism, and its drug susceptibility are analyzed.

## 2. Results

A total of 36 neonates were found to be colonized or infected with *R. mannitolilytica*: 18 colonized in 2020 and 13 colonized and 5 infected in 2021 (Table 1). Most colonized/infected patients were on mechanical ventilation, had vascular accesses (mainly central venous catheters), and nearly all received broad-spectrum antibiotics prior to colonization/infection with *R. mannitolilytica*. The patients’ characteristics and risk factors are presented in Table 2.

Among the colonized patients, 18 were colonized in the gastrointestinal tract (positive rectal swabs), 8 in the respiratory tract (positive nasal swabs and/or miniBAL), and 5 were colonized at both sites. The median time for gastrointestinal tract colonization was 13 days of NICU stay, whereas, for respiratory tract colonization, it was 21 days.

Two out of five infected patients had prior colonization of the gastrointestinal tract: one was diagnosed with a surgical site infection (without bacteremia), while the other developed a bloodstream infection. Another patient, with prior colonization of the respiratory tract, developed pneumonia associated with mechanical ventilation (without bacteremia). Two patients developed infections—a bloodstream infection and a urinary tract infection (without bacteremia)—without any prior colonization detected.

None of the patients died as a result of *R. mannitolilytica* infection. All the infected neonates were successfully treated with targeted therapy—four with ciprofloxacin, and one with bloodstream infection received a combination of ciprofloxacin and piperacillin-tazobactam.

The susceptibility to carbapenems (imipenem, meropenem), amikacin, fluoroquinolones (ciprofloxacin, levofloxacin), and sulfamethoxazole-trimethoprim (TMP-SMX) was determined for 11 *R. mannitolilytica* isolates (five infecting and six colonizing strains). Additionally, depending on the patients’ condition, the susceptibility of the selected isolates to ceftazidime (n = 7), cefepime (n = 4), tobramycin (n = 10), gentamicin (n = 9), and piperacillin-tazobactam (n = 5) was tested. All the isolates tested were resistant to carbapenems and aminoglycosides, whereas all were susceptible to TMP-SMX, and the vast majority were susceptible to fluoroquinolones. All the infected patients had received meropenem and/or gentamicin prior to the *R. mannitolilytica* infection. Detailed susceptibility results are presented in Table 3, and the minimum inhibitory concentrations (MICs) of the tested drugs are shown in Table 4.

Once an increased number of *R. mannitolilytica* isolates was observed in mid-2020, an epidemiological investigation was initiated. This included the creation of line-lists, environmental testing, and direct observation of infection control (IC) practices in the ward by the IC team. Although no breaches in IC protocols were identified, additional refresher trainings on IC measures, including hand hygiene, were conducted. An attempt was made to isolate or cohort colonized/infected patients; however, this was not always feasible due to a limited number of isolation rooms. In such cases, bedside isolation was implemented.

The first environmental testing, conducted in June 2020, revealed a potential source of *R. mannitolilytica*: the water heating system used to warm feeding bottles and nasogastric tube feeding formula, where abundant growth of *R. mannitolilytica* was detected (>1000 CFU/mL). A single heater was used for all NICU patients, and it was located in a formula preparation room with access restricted to staff only. Initially, the heater was filled with sterile water supplied in 5-L containers prepared by the hospital pharmacy. Once it was identified as the source of *R. mannitolilytica*, sterile water from disposable 1-L bottles was introduced. Initial attempts to sterilize the heater involved draining, disinfecting all surfaces, and heating freshly poured sterile water in the heater tank to the maximum temperature (95 °C). This procedure, performed daily, was only temporarily effective. After a few weeks, cultures from the heater tested positive for *R. mannitolilytica* again, leading to its replacement with a water-free system.

Unfortunately, eliminating this source did not lead to the cessation of the outbreak. Environmental testing continued, but no additional reservoirs were identified for an extended period. Finally, in October 2021, a new source was discovered: a water bath used for warming bottles of sterile water for patient bathing. This water bath was kept in a storage room away from the main ward facility, which allowed it to remain unnoticed by the infection control team for a long time.

## 3. Discussion

*Ralstonia* spp. are environmental microorganisms commonly found in soil, water, and on plant surfaces [5,6]. Certain species have also been recognized as opportunistic human pathogens, capable of causing a wide range of infections, including bacteremia, endocarditis, infections of bones, bone marrow, and joints, and infections linked to invasive medical procedures such as hemodialysis or surgery [10,11,12,13,14,15,16,17,18]. In neonates, *Ralstonia* spp. has been associated with bacteriemia and late-onset sepsis [3,4,7]. In our patients, despite bloodstream infections, cases of pneumonia associated with mechanical ventilation, surgical site infections, and urinary tract infections without concomitant bacteriemia were also diagnosed.

*Ralstonia* spp. can contaminate medical equipment and fluids used in medical procedures, including those with direct access to the bloodstream [7,19,20]. This may be caused by the contamination of fluids at the production stage, as there are reports of *Ralstonia* spp. microorganisms penetrating bacteriological filters with pores of 0.45 and 0.22 µm in diameter [5]. *Ralstonia* spp. has also been reported to cause infections associated with the use of heated liquids (the optimum temperature for *R. mannitolilytica* growth is 30 °C to 37 °C, but it can also grow at 42 °C) [5,21].

Patients in NICUs, due to numerous risk factors, are particularly prone to colonization and/or infections with various microorganisms, including opportunistic pathogens [22]. The majority of our patients were preterm neonates, hospitalized in the NICU for a long time, and subjected to invasive procedures such as mechanical ventilation and/or central venous catheterization. Additionally, nearly all patients included in this study had received broad-spectrum antibiotics because of proven or suspected infections during the course of the NICU stay. The latter, leading to the disturbance of neonatal microbiota, may be of special significance, since human microbiota is crucial for protecting against colonization by pathogenic and resistant microorganisms [23,24].

Since NICU patients represent a highly vulnerable population, stringent infection control measures are of utmost importance in these settings. Despite our efforts (such as routine surveillance cultures on admission, isolation of colonized/infected patients, hand hygiene, use of disposable equipment, etc.), we were unable to prevent and quickly contain an outbreak of *R. mannitolilytica* in our NICU. One possible explanation for this situation is the overwhelming impact of the COVID-19 pandemic, which demanded the full attention and efforts of medical staff at the time. In the early months of 2020, our primary focus was on implementing strict infection control measures to prevent the spread of SARS-CoV-2. These measures included reorganizing patient admissions and ward layouts to minimize crowding, establishing dedicated subunits for infected or suspected patients with assigned personnel, introducing universal PCR screening for all patients and their caregivers upon admission, conducting surveillance PCR testing of medical staff, and ensuring an adequate supply of personal protective equipment and disinfectants. Although our hospital did not experience shortages of personnel, equipment, or disinfectants, the infection control team’s intense focus on COVID-19—along with staff anxiety about contracting the virus—diverted attention from other potential threats. Studies have reported that during COVID-19 surges, bacterial healthcare-associated infections increased, suggesting that the heightened focus on COVID-19 may have inadvertently led to lapses in routine infection prevention and control practices, allowing other pathogens to spread [25]. As a result, the initial isolations of *R. mannitolilytica* went unnoticed, and the search for environmental reservoirs was not fully successful at the beginning of the epidemiological investigation. This phenomenon is not unique to our experience; other authors have also reported challenges in identifying the source of *R. mannitolilytica* infections in hospital settings [26,27].

Eventually, the source of colonization and infection of our patients turned out to be the water heater for bottles with the patients’ feeding formula and the water heater for bottles with sterile water for bathing. Although the contents of the heated containers were free from microorganisms (feeding bottles and bottles with bath water), microbial contamination of their outer surfaces was enough to lead to patients’ colonization and/or infection with *R. mannitolilytica*. The use of medical devices such as vascular accesses, nasogastric tubes, and mechanical ventilation are known to facilitate this process [28,29]. The failure to eradicate *Ralstonia* spp. from the heater chamber was most likely due to its ability to form biofilms on various materials (including steel surfaces). The biofilm is difficult to remove and allows microorganisms to survive in hostile conditions, such as exposure to disinfectants or nutrient deficiency [30,31]. The outbreak was ultimately controlled by removing the water heaters from the ward and replacing them with water-free systems. An alternative approach could have been to provide personalized equipment, such as individual bottle heaters. However, this option was not considered due to its limited feasibility in our setting, as managing multiple individual heaters would have been challenging.

Water has increasingly been recognized as a source of multidrug-resistant microorganism outbreaks in hospitals, particularly in intensive care units [32]. As a result, there is growing interest in implementing water-free environments in such settings. Several interventions have been proposed to effectively reduce the risk of infection and colonization by waterborne microorganisms in ICUs. These include the removal of sinks from patient care areas, the use of waterless bathing products, and bottled water for drinking, oral care, and medication preparation [33]. In the NICU, removing sinks and modifying bathing practices—such as performing baths at the bedside using bottled sterile water and disposable wipes—has been shown to contain prolonged outbreaks caused by multidrug-resistant Gram-negative bacteria [34]. Additional interventions in the NICU may include using wipes to clean incubator surfaces, reducing incubator humidification to prevent excessive condensation, and employing ultraviolet sterilization for milk bottles [35].

Relatively little is known about *R. mannitolilytica* susceptibility, and various susceptibility profiles have been reported in the literature [26]. There are no EUCAST breakpoints for this microorganism, so varying criteria have been applied by different authors [18]. In our study, EUCAST PK/PD (non-species-specific) criteria (valid for 2020/2021) were applied for all drugs except trimethoprim-sulfamethoxazole, for which no such criteria were available. Instead, susceptibility was assessed using the criteria established for other non-fermenting rods, i.e., *Acinetobacter* spp.

All the tested isolates were resistant to carbapenems and aminoglycosides, likely due to prior exposure of the patients to these agents. *R. mannitolilytica* is believed to easily gain resistance, and broad antibiotic therapy is an important risk factor for resistance [36]. Similarly to previous reports, all our isolates were susceptible to TMP-SMX, and the vast majority were susceptible to fluoroquinolones [26]. This may be associated with the rare use of these drugs in the neonatal population.

In previous studies, various *Ralstonia* species, primarily *R. mannitolilytica* and *R. pickettii*, have consistently exhibited high resistance to colistin, aztreonam, and aminoglycosides [6,37]. However, susceptibility to β-lactams, including carbapenems, has been more variable. Genetic studies have shown that *Ralstonia* isolates harbor OXA-22 and/or OXA-60 family ß-lactamases genes, which contribute to the high MICs of β-lactam antibiotics [27,31,37,38,39]. However, the distribution of specific genes from these families (i.e., *blaOXA-22*, *blaOXA-443*, *blaOXA-569*, *blaOXA-572*, *blaOXA-574*, *blaOXA-899*, *blaOXA-444*, *blaOXA-570*, *blaOXA-571*, *blaOXA-573*, *blaOXA-60*, *blaOXA-898*) varies among species and individual strains [38]. Among these, *blaOXA-572*, *blaOXA-571*, *blaOXA-444,* and *blaOXA-443* are the most frequently detected in *R. mannitolilytica* [38]. The last two genes have been found in carbapenem-resistant *R. mannitolilytica* isolates [27,38,39]. Multidrug resistance in *Ralstonia* spp. can be associated with the presence of multidrug efflux pumps. Whole genome sequencing has revealed the presence of multidrug efflux pumps genes in many *Ralstonia* spp. isolates, including the *ceoB* gene encoding a component of CeoAB-OpcM, which conquers resistance to fluoroquinolones and aminoglycosides [38]. The *ceoB* gene has been found in most *R. mannitolilytica* isolates [38]. Aminoglycoside resistance often results from enzymatic inactivation. However, the frequency and distribution of specific genes encoding aminoglycoside-modifying enzymes varies in *Ralstonia* spp. Genes such as *aac(3)-IVa*, *aadA2*, *ant(2″)-la*, *aph(3″)-lb*, *aph(4)-la,* and *aph(6)-ld* have been detected in some *R. mannitolilytica* strains [37,38].

Generally, most *Ralstonia* strains remain susceptible to TMP-SMX and ciprofloxacin; however, susceptibility can differ depending on the origin of the isolates. Although rare, resistance to sulfonamides in *R. mannitolilytica* has been associated with the presence of the *sul1* gene, which encodes a dihydropteroate synthase that restores folic acid metabolism in bacterial cells [38]. Notably, *R. mannitolilytica* strains recovered from cystic fibrosis patients have shown significantly higher MICs for ciprofloxacin and trimethoprim-sulfamethoxazole, likely due to prolonged antibiotic exposure in this patient group [37]. These variations underscore the importance of considering both the bacterial species and clinical context when assessing *Ralstonia* susceptibility.

Based on our findings, TMP-SMX and fluoroquinolone appear to be the most active drugs for empirical treatment when *R. mannitolilytica* infection is suspected. Nonetheless, due to the potential for asymptomatic colonization by this microorganism and the adverse effects of antimicrobial agents—such as toxicity, resistance development, and disruption of the microbiota—antibiotic use in NICU settings must be highly judicious and restricted to cases where infection is confirmed or strongly suspected. All our infected patients were successfully treated with targeted therapy using ciprofloxacin (with one additionally receiving piperacillin-tazobactam), and no drug-related hematological, hepatic, or renal disturbances were observed. This aligns with previous studies indicating that ciprofloxacin is safe even in preterm neonates and does not cause long-term growth impairment [40].

Future research should focus on more effective methods for identifying and eradicating environmental reservoirs of opportunistic pathogens, including addressing the challenges posed by biofilm formation on medical equipment. Additionally, standardizing the susceptibility testing criteria for *Ralstonia* spp. and investigating novel therapeutic options are crucial to improve the management of infections, particularly in the vulnerable neonatal population.

This study highlights the increasing clinical impact of *R. mannitolilytica* as an opportunistic pathogen in NICUs, emphasizing its ability to cause serious infections in vulnerable patients. The findings demonstrate the importance of contaminated water sources and invasive procedures in pathogen transmission, further complicated by significant antibiotic resistance. Addressing these challenges requires strict infection control and careful antimicrobial stewardship to prevent outbreaks and protect neonatal patients.

The microbiology laboratory plays a key role in the early detection of *R. mannitolilytica*, identifying colonization and infections through rapid culture analysis and susceptibility testing. Close collaboration with clinicians allows for timely recognition of resistant strains, guiding appropriate antibiotic therapy and infection control measures. This partnership is essential for limiting the spread of multidrug-resistant *Ralstonia* spp. in NICUs and improving patient safety.

## 4. Materials and Methods

After the notification of an increasing number of *R. mannitolilytica* isolations from neonates hospitalized in the neonatal intensive care unit (NICU) in mid-2020, we initiated an epidemiological investigation. Clinical and microbiological records of all the neonates hospitalized in the NICU during the outbreak were analyzed, and possible environmental sources of *R. mannitolilytica* and modes of transmission were sought.

Patients were diagnosed with colonization and/or infections with *R. mannitolilytica* based on clinical symptoms and microbiological results, i.e., rectal and nasal swabs, miniBAL, blood, urine, and wound cultures.

Blood cultures were incubated using the BD Bactec FX 200 system (Becton Dickinson, Franklin Lakes, NJ, USA). Once a growth signal was detected, the bottles were subcultured onto solid Columbia agar with 5% sheep blood and chocolate agar (Graso, Starogard Gdański, Poland). These plates were incubated for up to 48 h at 37 °C in an aerobic atmosphere enriched with 5% CO_2_. miniBAL samples were collected in sterile containers and quantitatively cultured on solid Columbia agar with 5% sheep blood and chocolate agar (Graso). The plates were incubated for up to 48 h at 37 °C in an aerobic atmosphere enriched with 5% CO₂. Wound swabs collected using Amies transport medium (Copan, Murrieta, CA, USA) were cultured on solid Columbia agar with 5% sheep blood, chocolate agar, MacConkey agar, and in liquid BHI broth (Graso). These were incubated for up to 48 h at 37 °C in an aerobic atmosphere enriched with 5% CO_2_. Urine samples were quantitatively cultured on solid Columbia agar with 5% sheep blood and UriSelect4 medium (BioRad, Hercules, CA, USA) and incubated for up to 24 h at 35 °C in an aerobic atmosphere. Nasal and rectal swabs for the detection of multidrug-resistant pathogens were collected using Amies transport medium (Copan). The samples were cultured on solid Columbia agar with 5% sheep blood, MacConkey agar (Graso), and chromogenic media, including ChromID MRSA, ESBL, VRE, and CARBA (bioMérieux, Marcy-l’Étoile, France). Incubation lasted 18–24 h under aerobic conditions at 35 °C.

Environmental studies included water samples from bottle warmers, swabs from all equipment with direct patient contact, and surface swabs from areas in patients’ rooms. Water samples were cultured in liquid TSB medium (Graso) at a 1:10 ratio and incubated for up to three days, with daily observation for turbidity. Positive broth cultures were subcultured on solid Columbia agar with 5% sheep blood and incubated for up to 48 h under aerobic conditions at 35 °C. Swabs were cultured on solid Columbia agar with 5% sheep blood and in liquid TSB medium, incubated for up to 48 h under aerobic conditions at 35 °C.

Identification of cultured microorganisms was performed using a matrix-assisted laser desorption-ionization time-of-flight (MALDI-TOF) Biotyper (Bruker Daltonics, Bremen, Germany). Drug susceptibility was conducted using the antibiotic concentration gradient strip method (E-test, bioMérieux), following the manufacturer’s instructions. The results were interpreted according to EUCAST recommendations (versions 10 and 11), using PK-PD (non-species-related) breakpoints. For trimethoprim-sulfamethoxazole, the criteria for *Acinetobacter* spp. were applied. The following MIC values (mg/L) were used for resistance categorization: ceftazidime > 8; cefepime > 8; piperacillin-tazobactam > 16; imipenem > 4; meropenem > 8; amikacin > 1; gentamicin > 0.5; tobramycin > 0.5; ciprofloxacin > 0.5; levofloxacin > 1; trimethoprim-sulfamethoxazole > 4.

For each patient included in the analysis, specific factors and procedures that could serve as potential risk factors for colonization or infection were identified. These factors included gestational age at birth, length of stay in the NICU, use of mechanical ventilation, presence of vascular access devices, mode of nutrition (parenteral, nasogastric tube, feeding bottle), and administration of antibiotic therapy.

## 5. Conclusions

This study highlights the critical role of environmental water reservoirs and invasive procedures in the colonization and infection of NICU patients with *R. mannitolilytica*. Effective outbreak management requires robust infection control measures, early identification and elimination of environmental sources, and judicious antibiotic use to mitigate microbiota disturbances and the development of resistance. Enhanced surveillance and biofilm-targeted interventions, particularly the elimination of water reservoirs, are essential to prevent similar outbreaks in high-risk settings.

## Figures and Tables

**Table 1:** *Ralstonia mannitolilytica* colonizations and infections.

	Gastrointestinal Tract Colonization Only	Respiratory Tract Colonization Only	Gastrointestinal and Respiratory Tract Colonization	Infection After Prior Colonization	Infection Without Prior Colonization
2020 (n = 18)	11	5	2	0	0
2021 (n = 18)	7	3	3	3	2
Total (n = 36)	18 (50%)	8 (22%)	5 (14%)	3 (8%)	2 (6%)

**Table 2:** Patients’ characteristics, selected risk factors, and time to colonization.

Gender, female/male (%)	14/22 (39/61)
Gestational age at birth (weeks) *	34 (22–40)
Birth weight (g) *	2220 (480–4500)
Mechanical ventilation (%)	26 (72)
Central venous catheter (%)	23 (64)
Prior broad-spectrum antibiotic therapy (%)	33 (92)
Mode of feeding (%):	
parenteral	20 (56)
nasogastric tube	7 (19)
feeding bottle	9 (25)
Time to the gastrointestinal tract colonization (days at NICU) *	13 (4–83)
Time to the respiratory tract colonization (days at NICU) *	21 (1–102)

* median and range.

**Table 3:** *Ralstonia mannitolilytica* antibiotic resistance.

Antibiotic (no of tested strains)	CAZ	FEP	TZP	IPM	MEM	AN	GM	TM	CIP	LVX	SXT	MDR
	(n = 7)	(n = 4)	(n = 5)	(n = 11)	(n = 11)	(n = 11)	(n = 9)	(n = 10)	(n = 11)	(n = 11)	(n = 11)	
Number (%) of resistant strains	2	2	2	11	11	11	9	10	1	1	0	4
	(29)	(50)	(40)	(100)	(100)	(100)	(100)	(100)	(9)	(9)		(36)

CAZ: ceftazidime; FEP: cefepime; TZP: piperacillin-tazobactam; IMP: imipenem; MEM: meropenem; AN: amikacin; GM: gentamicin; TM: tobramycin; CIP: ciprofloxacin; LVX: levofloxacin; SXT: trimethoprim-sulfamethoxazole; MDR: multidrug resistant, defined as acquired non-susceptibility to at least one agent in three or more antimicrobial categories.

**Table 4:** Minimum inhibitory concentrations of antibiotics for *Ralstonia mannitolilytica* isolates.

Isolate No	MIC (mg/L)										
	CAZ	FEP	TZP	IMP	MEM	AN	GM	TM	CIP	LVX	SXT
1 *	2	nd	nd	6	32	256	48	24	0.75	2	0.032
2	4	nd	nd	12	32	256	64	8	0.125	0.25	0.094
3	4	nd	8	12	32	256	24	nd	0.125	0.25	0.094
4 *	32	8 *	64	16	16	16	nd	8	0.125	0.25	0.125
5 *	3	8 ^	4	16	16	16	8	8	0.094	0.19	0.125
6 *	32	2	64	16	16	32	8	16	0.25	0.25	0.125
7	nd	nd	nd	16	16	16	8	8	0.125	0.25	0.125
8	6	1.5	8	8	32	16	nd	8	0.125	0.25	0.064
9	nd	nd	nd	16	16	16	8	8	0.125	0.38	0.047
10	nd	nd	nd	16	16	16	8	8	0.125	0.25	0.125
11	nd	nd	nd	16	16	32	8	16	0.125	0.25	0.125

CAZ: ceftazidime; FEP: cefepime; TZP: piperacillin-tazobactam; IMP: imipenem; MEM: meropenem; AN: amikacin; GM: gentamicin; TM: tobramycin; CIP: ciprofloxacin; LVX: levofloxacin; SXT: trimetho-prim-sulfamethoxazole. nd: not done. * Strains with multidrug resistant phenotype identified. ^ Due to the threshold MIC value, the strain was classified as resistant to cefepime.

## Data Availability

The data presented in this study are available on request from the corresponding author.

## Abbreviations

The following abbreviations are used in this manuscript:

MDPI	Multidisciplinary Digital Publishing Institute
DOAJ	Directory of open access journals
TLA	Three letter acronym
LD	Linear dichroism
